# Multidimensional roles and clinical significance of GATA3 in breast cancer

**DOI:** 10.3389/fcell.2026.1919039

**Published:** 2026-09-01

**Authors:** Junjuan Tan, Weihan Chen, Kaiyan Song, Xiaoqian Zhu, Liwei Hong, Juju Zhou, Kai Chen, Haitong Lv

**Affiliations:** 1 Cancer Hospital of Shantou University Medical College, Shantou, China; 2 Yangzhou University Faculty of Medicine, Yangzhou, China; 3 The First People’s Hospital of Foshan, Foshan, Guangdong, China; 4 Shantou Central Hospital, Shantou Clinical Medical College of Jinan University, Shantou, China

**Keywords:** breast cancer, diagnostic biomarker, GATA3, prognosis, transcription factor, TRPS1, tumor microenvironment

## Abstract

**Background:**

GATA-binding protein 3 (GATA3) is a pivotal transcriptional regulator during mammary gland development and serves as a core biomarker in breast cancer. Although traditionally recognized for maintaining luminal differentiation, its high mutation frequency reveals a complex, dual role in tumorigenesis.

**Methods:**

This review summarizes the multidimensional roles of GATA3, with an emphasis on molecular regulatory networks, clinical diagnostic applications, and significance in therapeutic response in the field of breast cancer.

**Results:**

At the molecular level, GATA3 operates within a core transcriptional regulatory network, synergistically interacting with key factors like ERα and FOXA1 to govern the luminal phenotype. Clinically, GATA3 serves as a highly sensitive core biomarker for distinguishing between primary and metastatic breast cancer. Notably, emerging biomarkers like TRPS1 and SOX10 provide essential complementary value in the diagnosis of triple-negative breast cancer. High expression of GATA3 strongly correlates with a favorable prognosis and sensitivity to endocrine therapy. Conversely, downregulated GATA3 expression enhances tumor aggressiveness, increases sensitivity to certain chemotherapeutic agents such as anthracyclines, and promotes the formation of responsive “hot” microenvironment. Furthermore, although GATA3 mutations are relatively common among patients with breast cancer, their clinical and prognostic significance warrants further in-depth investigation.

**Conclusion:**

As a core regulator of breast cancer cell plasticity and the tumor microenvironment, GATA3 extends far beyond its traditional role as a luminal biomarker. Elucidating its multidimensional roles and exploring its emerging therapeutic potential will continue to propel the field of breast cancer toward more sophisticated and personalized diagnostic and therapeutic paradigms.

## 1 Introduction

**FIGURE 1:**
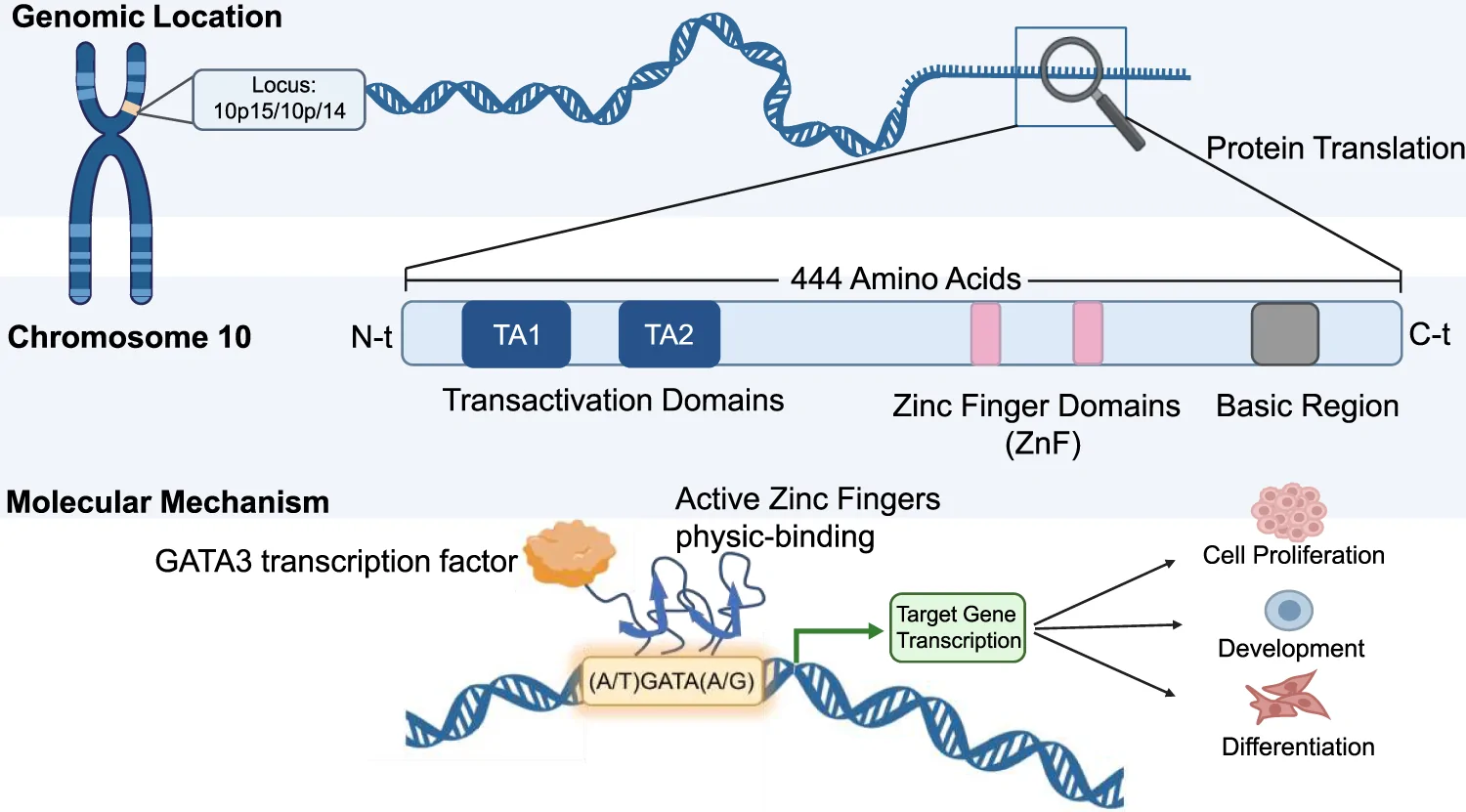
Structure and Transcriptional Regulation Mechanism of GATA3. The upper panel illustrates the genomic locus of human GATA3 on chromosome 10 and its protein architecture, which consists of two transactivation domains (TA1, TA2), two zinc-finger domains (ZnF), and a C-terminal basic region, containing a total of 444 amino acids. The lower panel depicts the core molecular mechanism: the GATA3 transcription factor binds to the consensus DNA sequence (A/T) GATA (A/G) via active zinc-finger motifs, triggering target-gene transcription and further regulating cell proliferation, development, and differentiation.

GATA-binding protein 3 (GATA3) is a highly conserved zinc finger transcription factor essential for mammary luminal epithelial cell differentiation. Conditional knockout studies confirm that GATA3 loss abolishes luminal fate commitment in mouse mammary glands (Kouros-Mehr et al., 2006), establishing its role as a master regulator of lineage identity. In breast cancer, GATA3 has evolved from a developmental factor into a molecule with multifaceted clinical significance—functioning as a diagnostic marker for mammary origin, a prognostic indicator, and a determinant of therapeutic response (Figure 1).

GATA3 expression is tightly linked to the luminal subtype and ERα positivity (Martin et al., 2021), and its immunohistochemical detection demonstrates superior sensitivity over traditional markers in identifying metastatic breast cancer. High expression correlates with well-differentiated tumors and favorable outcomes (Re et al., 2023), while its loss—which may occur through promoter hypermethylation, inactivating mutations, or post-translational degradation—is associated with basal-like transformation, increased aggressiveness, and poor prognosis (Saele et al., 2025). Crucially, however, GATA3 mutations rank among the most frequent somatic alterations in breast cancer, and emerging evidence reveals that distinct mutation classes exert fundamentally opposing prognostic effects (Medford et al., 2025). This duality—simultaneously a “guardian” of differentiation and a potential oncogenic “driver”—remains a central unresolved controversy. Therapeutically, GATA3 profoundly influences the sensitivity and development of resistance to endocrine therapy through its interactions with key signaling pathways such as the ERα and is also associated with chemotherapy response and the shaping of the tumor immune microenvironment (Martin et al., 2021). While previous reviews have addressed individual aspects of GATA3 biology in breast cancer, most have focused predominantly on a single dimension, such as its prognostic value (Guo et al., 2017) or its role as a prognostic factor limited to less aggressive subtypes (Querzoli et al., 2021). However, none has systematically integrated the transcriptional regulatory network, the evolving diagnostic landscape incorporating novel markers such as TRPS1 and SOX10, the prognostic implications of both expression levels and distinct mutation classes, and the role of GATA3 in modulating responses to endocrine therapy, chemotherapy, and immunotherapy within a single framework. This article aims to systematically review the biological functions, molecular regulatory networks, clinical applications, and mutational characteristics of GATA3 in breast cancer, providing a theoretical basis for deepening the understanding of this disease and advancing personalized diagnosis and treatment.

## 2 The GATA3 transcriptional regulatory network in breast cancer

### 2.1 The ERα-FOXA1-GATA3 core axis and extended interactions

As a pioneer transcription factor, GATA3 binds nucleosomal DNA and remodels chromatin to enable cooperative factor recruitment (Pavithran and Kumavath, 2021). In breast cancer, its high expression defines the luminal subtype (Luminal A and B), maintaining luminal differentiation characteristics including ERα and cytokeratin 8/18 expression through direct activation of target gene promoters and enhancers (Porras et al., 2022). Conversely, GATA3 loss triggers dedifferentiation toward basal-like characteristics (CK5/6 expression), epithelial-mesenchymal transition (EMT), and enhanced invasiveness (Saele et al., 2025), positioning GATA3 as a lineage-specific “master switch” whose functional integrity suppresses malignant progression.

The transcriptional activity of GATA3 is primarily realized through a tripartite regulatory network with ERα and FOXA1. These three factors co-bind enhancer regions and cooperatively regulate thousands of target genes driving luminal breast cancer growth (Kumar and Vindal, 2023). Within this hierarchy, FOXA1 opens compacted chromatin to create binding sites for ERα and GATA3, while GATA3 stabilizes the complex and modulates ERα transcriptional output (Pavithran and Kumavath, 2021). This synergy forms the molecular basis for endocrine dependence and exhibits tissue specificity—*cBioPortal* analysis reveals a strong positive correlation among these three factors in breast cancer that is absent in ovarian and endometrial cancers. Notably, GATA3 directly binds the ERα gene enhancer to upregulate its transcription, while ERα in turn activates GATA3 expression, forming a self-reinforcing positive feedback loop that sustains luminal identity (Grindeland et al., 2025). Beyond this core triad, GATA3 interacts with the orphan nuclear receptor NR2C2 to regulate tumor progression-associated transcription programs (Plagens et al., 2025), and is subject to post-translational regulation through AKT-mediated phosphorylation, which triggers 14-3-3τ recognition and proteasomal degradation (Garan et al., 2022).

### 2.2 Epigenetic regulation and phase separation

GATA3 is regulated at the epigenetic level through DNMT3A-driven promoter hypermethylation, an important silencing mechanism in aggressive breast cancers (Vitte et al., 2025). A recent discovery reveals that GATA3 forms transcriptional condensates through liquid-liquid phase separation, with the second zinc finger domain playing a critical regulatory role (Chen et al., 2025). This finding provides a mechanistic framework linking cancer-specific mutations to their diverse transcriptomic consequences—mutations disrupting phase separation differentially reshape gene expression programs depending on their location within the protein. The metabolic-epigenetic axis further intersects with GATA3 regulation: 2-hydroxyglutarate, an oncometabolite accumulated in IDH-mutant tumors, disrupts GATA3 motif accessibility, while the pyruvate kinase M2 (PKM2)-EZH2 axis modulates H3K27Ac at the GATA3 promoter, collectively determining luminal lineage fidelity (Yomtoubian et al., 2020).

### 2.3 Non-coding RNA regulatory circuits

**FIGURE 2:**
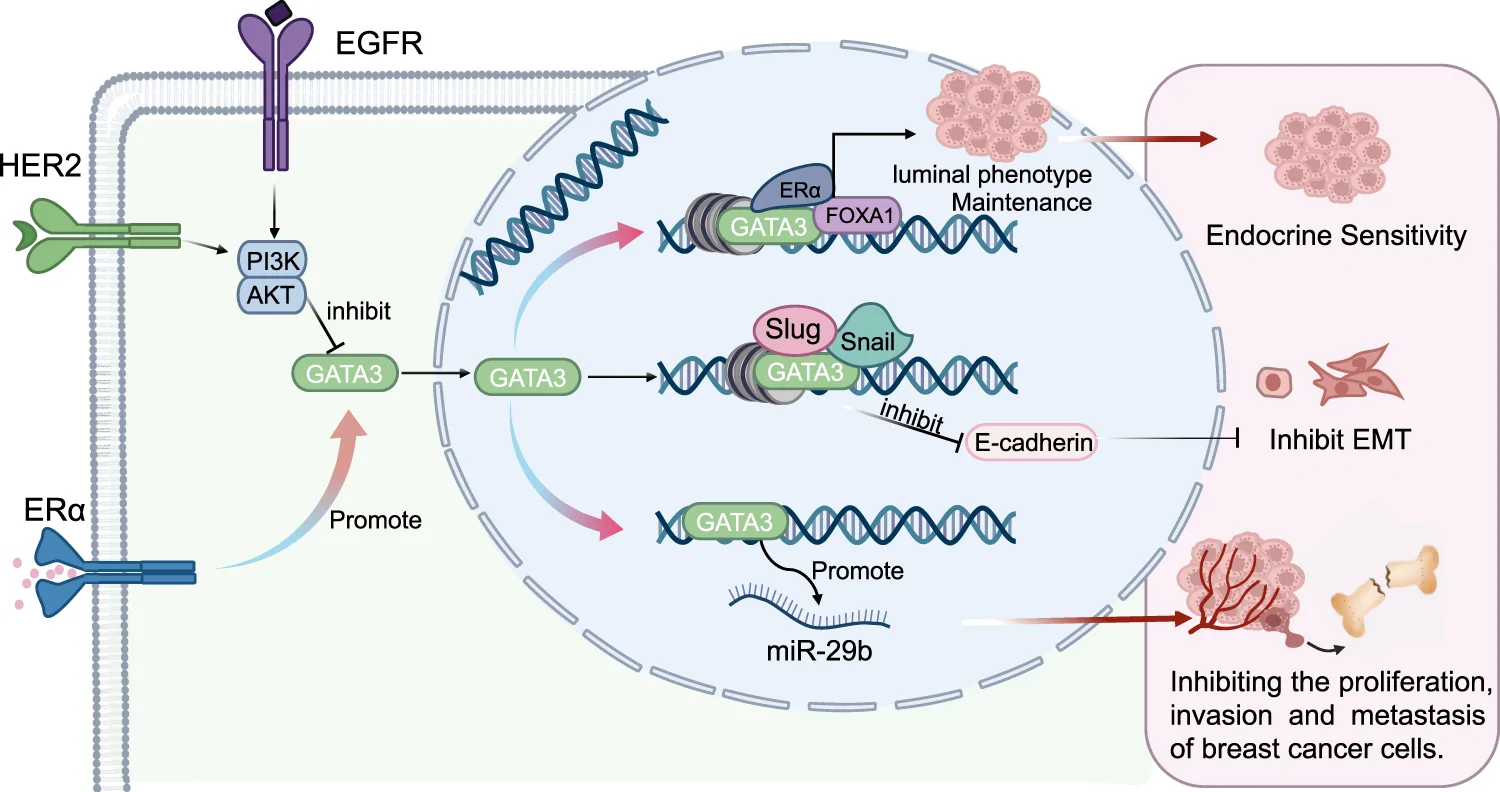
The Molecular Regulatory Network of GATA3 in Breast Cancer. Extracellular signals mediated by EGFR-PI3K-AKT and HER2 suppress GATA3 expression, whereas ERα promotes GATA3 expression. Inside the nucleus, GATA3 cooperates with ERα and FOXA1 to maintain luminal phenotype and preserve endocrine sensitivity. Meanwhile, GATA3 represses Slug- and Snail-driven EMT by up-regulating E-cadherin. Furthermore, GATA3 induces the transcription of miR-29b, which ultimately restrains breast-cancer-cell proliferation, invasion and metastasis.

Multiple non-coding RNA species participate in GATA3 regulation. Upstream, circular RNAs such as circ_0000977 act as miRNA sponges (e.g., adsorbing miR-135b-5p) to indirectly modulate GATA3 expression (Darbeheshti et al., 2023). Downstream, GATA3 transcriptionally activates miR-29 b and miR-205–5p, which target genes involved in proliferation and metastasis, exerting tumor-suppressive effects (Amirian et al., 2023). GATA3 also maintains the epithelial phenotype by regulating transcriptional repressors Snail and Slug, preserving E-cadherin expression and inhibiting EMT (Ashaie and Chowdhury, 2016). The GATA-type zinc finger transcription factor TRPS1 participates in parallel regulatory circuits, influencing phenotypic transformation through the miR-221/222-E-cadherin axis (Bao et al., 2013). Together, these multi-layered interactions—spanning transcription factor cooperativity, epigenetic modifications, phase separation dynamics, and non-coding RNA circuits—constitute the regulatory architecture shaping luminal identity and biological behavior in breast cancer cells (Figure 2). Table 1 (Pavithran and Kumavath, 2021; Grindeland et al., 2025; Plagens et al., 2025; Garan et al., 2022; Vitte et al., 2025; Yomtoubian et al., 2020; Darbeheshti et al., 2023; Amirian et al., 2023; Bernard and Goldstein, 2024; Pantelaiou-Prokaki et al., 2022; Tan et al., 2025; Xu et al., 2020; Erdős and Bálint, 2020; Takaku et al., 2020; Kusi et al., 2022; Darbeheshti et al., 2021).

**TABLE 1:** Core functions and molecular regulatory mechanisms of GATA3.

Functional category	Biological role	Mechanism	References
Cell differentiation and tumor suppressive function	Maintenance of the luminal phenotype	• GATA3 and FOXA1 promote ESR1 binding via chromatin remodeling, activate luminal genes to maintain the luminal phenotype and suppress tumors	Pavithran and Kumavath (2021)
		• ERα, GATA3, and FOXA1 are positively correlated and cooperate to drive cancer progression by regulating the genomic localization of ERα	Grindeland et al. (2025)
		• FOXA1/GATA3 complex co-occupies a subset of genomic regions to drive transcriptional programs involved in tumor progression	Plagens et al. (2025)
	Inhibition of tumor invasion	• GATA3, Snail and FOXA2 cooperatively regulate the transcription of E-cadherin, a key EMT hallmark, thereby controlling breast cancer cell invasion and metastasis.	Ashaie and Chowdhury (2016)
		• GATA3-associated epithelial cell-specific differentiation regulator TRPS-1 is targeted by miR221/222. This targeting suppresses E-cadherin, thereby promoting EMT and tumor invasion/metastasis	Bao et al. (2013)
Molecular regulatory networks	Modulation of ERα signaling	GATA3 and ERα form a direct positive feedback loop, amplifying each other's expression and activity, thereby driving continuous oncogenic signaling and tumor proliferation	Xu et al. (2020)
	Epigenetic modifications	GATA3 is transcriptionally silenced by aberrant activation of DNMT3B-mediated promoter hypermethylation, which promotes tumor progression	Vitte et al. (2025)
	Interaction with non-coding RNAs	• GATA3 is regulated by circ_0000977 and circ_0044234 via a miR-135b-5p-dependent ceRNA mechanism	Darbeheshti et al. (2021), Darbeheshti et al. (2023)
		• GATA3 is regulated by miR-29 family, thereby defining a GATA3-miR-29b regulatory axis that suppresses cell proliferation, invasion, and metastasis	Amirian et al. (2023)
Diagnosis and prognosis value	Distinguishing primary and metastases	• GATA3 is a key immunohistochemical marker that distinguishes primary from metastatic breast cancer based on differential expression: highly expressed in primary tumors, but low or lost in metastases	Sangoi et al. (2016)
	Subtypes identification	GATA3 expression is subtype-dependent in breast cancer: high in luminal and HER2+ subtypes but weak in TNBC	Lui et al. (2024)
​	Clinical outcomes prediction	• Increased 2-hydroxyglutarate impairs chromatin accessibility of GATA3, drives luminal-to-basal-like expression shift, and increases tumor heterogeneity associated with poor breast cancer prognosis	Kusi et al. (2022)
		• GATA3 expression level is a prognostic biomarker, with high levels indicating favorable outcomes (low histological grade, positive hormonal receptors, and low Ki-67), whereas low GATA3, especially in TNBC, predicts a poorer prognosis	Bai et al. (2025), Yildirim et al. (2020)
Tumor microenvironment and therapy relevance	TME remodification	• In TNBC, GATA3 is epigenetically regulated by EZH2. EZH2 inhibition derepresses GATA3, reprogrammes basal-like tumour cell phenotype, and modulates tumour biological behaviour associated with treatment sensitivity	Yomtoubian et al. (2020)
		• Wang G et al. demonstrated that, GATA3 regulates proliferation-related dependent genes, facilitates an immunosuppressive microenvironment in breast cancer	Wang et al. (2025), Warm et al. (2024)
Remodels the TME	Treatment sensitivity evaluation	• Akt-14-3-3τ-mediated degradation of GATA3 contributes to endocrine therapy resistance in ER+ breast cancer by altering ERα isoform balance	Garan et al. (2022)
		• GATA3 promotes doxorubicin resistance in breast cancer by inhibiting CYB5R2-mediated iron metabolism and ferroptosis. Clinically, high GATA3 expression is associated with poor response to doxorubicin-based neoadjuvant chemotherapy	Zhu et al. (2023)

## 3 GATA3 in diagnostic pathology: established utility and emerging complementary markers

### 3.1 Diagnostic performance of GATA3 for mammary origin

GATA3 is a cornerstone immunohistochemical marker for confirming breast origin in metastatic carcinomas. In hormone receptor-positive subtypes (Luminal A and Luminal B), positivity exceeds 90% (Stolnicu et al., 2020), and in metastatic breast cancer it reaches 95%—far surpassing mammaglobin (SCGB2A2; 78%) and GCDFP-15 (65%) at both mRNA and protein levels (Sangoi et al., 2016). GATA3 staining typically exhibits a strong, diffuse nuclear pattern, which is particularly advantageous in limited tissue specimens such as core needle biopsies and cytology smears. The consistency of GATA3 expression between primary and metastatic sites is notably high (Pearson r = 0.81), making it a reliable tool for tracking mammary-origin tumors across diverse metastatic destinations including lymph nodes, lung, liver (Atmış et al., 2025; Haboub et al., 2025; Lou et al., 2021), gastrointestinal tract (Wang et al., 2023a), and bladder (Zhang et al., 2024).

GATA3 is especially indispensable in the diagnosis of occult breast cancer, where initial presentation is metastatic disease and the primary lesion evades conventional imaging; in such cases, GATA3 positivity in biopsy tissue is essential for establishing mammary origin (Zhang et al., 2021). The marker also retains high expression in male breast cancer, extending its diagnostic utility (Gonzalez et al., 2013). Currently, GATA3 is widely recommended as a core confirmatory marker for carcinomas of unknown primary when breast origin is suspected.

A notable limitation is cross-reactivity with urothelial carcinoma, certain salivary gland tumors, and cutaneous squamous cell carcinoma, necessitating panel-based interpretation in specific differential diagnosis scenarios. In particular, combining GATA3 with uroplakin II and p63 enables reliable distinction: a GATA3+/uroplakin II−/p63− profile favors breast origin, whereas GATA3+/uroplakin II+/p63+ supports urothelial origin (Naik et al., 2023). When distinguishing breast cancer from lung adenocarcinoma—particularly when ER- or HER2-positive lung cancers create diagnostic confusion—a TTF-1/Napsin A/GATA3 panel is highly effective (Wang et al., 2023b). For ovarian or peritoneal serous carcinoma metastatic to the breast, combining GATA3 with PAX-8 and CA-125 resolves the differential (Wang et al., 2023b). In gastrointestinal contexts, breast cancer metastases to the stomach typically express GATA3, whereas gastric cancer metastases to the breast are GATA3-negative, providing a straightforward distinguishing criterion (Teixeira et al., 2021; Kamiya et al., 2025).

### 3.2 TRPS1 and SOX10: bridging the TNBC diagnostic gap

The sensitivity of GATA3 drops considerably in TNBC, though more than half of TNBC cases still retain expression (Jain et al., 2024). This diagnostic vulnerability has driven the identification of complementary markers, among which TRPS1 and SOX10 have accumulated the most compelling clinical evidence in recent years.

TRPS1 demonstrates high sensitivity across all breast cancer subtypes. Its positivity in TNBC—including metaplastic carcinoma—significantly surpasses that of GATA3 (Lui et al., 2024). Multiple comparative studies confirm that while TRPS1 and GATA3 perform comparably in hormone receptor-positive tumors, TRPS1 offers superior sensitivity in ER-negative and HER2-positive disease. Combined TRPS1/GATA3 application has been identified as the optimal strategy for confirming breast origin in ER-negative distant metastases (Zhang et al., 2025). A novel MGP/GATA3/TRPS1 panel achieves at least two positive markers in 93% of breast cancers, substantially improving diagnostic coverage (Du et al., 2022). However, MGP has not yet been incorporated into routine clinical practice and requires further validation before widespread adoption.

SOX10 fills a distinct niche, with expression concentrating in basal-like and secretory TNBC subtypes in a pattern negatively correlated with GATA3 (Jamidi et al., 2020). Combined SOX10/GATA3 testing achieves a 94.7% detection rate in TNBC (Liu et al., 2022). In rare subtypes where GATA3 performs poorly—such as adenoid cystic carcinoma, particularly the solid variant—TRPS1 and SOX10 show higher positivity rates (Warm et al., 2024). In metaplastic spindle cell carcinoma, however, GATA3 itself retains high sensitivity and specificity for differentiating it from other spindle cell lesions (Bradt et al., 2023). For cytological specimens including pleural effusions and fine-needle aspirates, GATA3 combined with TRPS1 provides optimal diagnostic accuracy; notably, TRPS1 demonstrates higher specificity than GATA3 when differentiating breast carcinoma from mesothelioma due to its negativity in mesothelial neoplasms (Lan et al., 2024; Baban et al., 2023).

An integrated panel incorporating GATA3, TRPS1, SOX10, and organ-specific markers (PAX8, TTF1, CDX2), interpreted alongside morphology and clinical history, represents a promising approach for maximizing diagnostic precision across the full breast cancer spectrum (Na et al., 2022). The development of consensus guidelines for panel composition and interpretation algorithms—particularly for metastatic TNBC and carcinomas of unknown primary—remains an important unmet clinical need.

## 4 Prognostic significance of GATA3 expression and mutations

### 4.1 Expression-based prognostic stratification

High GATA3 expression in breast cancer is generally regarded as a favorable prognostic indicator. A meta-analysis involving over 5,000 patients confirmed its predictive value for prolonged time to progression (Ding et al., 2022), and numerous studies have linked high GATA3 levels to lower histological grade, smaller tumor size, ER/PR positivity, and lower Ki-67 index (de Medeiros Souza et al., 2022). Notably, these associations are largely attributable to the strong correlation between GATA3 and the luminal phenotype; whether GATA3 expression retains independent prognostic significance after adjusting for molecular subtype remains incompletely established. This association is most prominent in Luminal A and B subtypes, where high GATA3 expression reflects maintained cellular differentiation. Imaging studies corroborate these findings: GATA3-positive tumors tend to exhibit less vascular distribution and regular borders on ultrasound and MRI, consistent with low-aggressiveness features (Bai et al., 2025). In early invasive breast cancer, high GATA3 expression (>95% cell positivity) correlates with hormone receptor positivity and low proliferative activity (de Medeiros Souza et al., 2022), and in the neoadjuvant chemotherapy setting, high expression is associated with superior distant relapse-free survival (Yildirim et al., 2020).

Conversely, low or absent GATA3 expression is a reliable marker of tumor dedifferentiation and aggressive behavior. Such tumors exhibit higher histological grade, larger size, and increased lymph node metastasis rates (Tabriz et al., 2023; Oda et al., 2023). Mechanistically, GATA3 loss derepresses the transcriptional repressors Snail and Slug, thereby activating EMT and promoting enhanced cancer stem cell properties, upregulation of proliferation-related gene sets, and hypoxia pathway activation (Saele et al., 2025; Ashaie and Chowdhury, 2016; Huang et al., 2021). Notably, GATA3 loss also correlates with upregulated immune checkpoint expression and T-cell activation within the tumor microenvironment—an alteration that, while reflecting increased aggressiveness, may simultaneously create opportunities for immunotherapy (Chen et al., 2024). At the molecular subtype level, combined detection of GATA3 and FOXA1 can assist in distinguishing prognostically favorable low-grade ER-positive tumors from high-grade, highly proliferative ER-positive subtypes (Huang et al., 2021), and GATA3-inclusive gene signatures have been used to construct molecular classification schemes predicting long-term recurrence risk after neoadjuvant chemotherapy (Huang et al., 2023).

### 4.2 The GATA3 mutation paradox

GATA3 mutations occur in approximately 8%–18.5% of breast cancers, are more prevalent in the HR+/HER2-subtype, and are considered driver events in tumorigenesis (Pei et al., 2025). The mutation landscape shows notable heterogeneity across clinical contexts: frequency is lower in East Asian patients (with associated poorer outcomes), higher in young patients with Luminal A tumors (Font et al., 2020), and differs markedly between histological types—approximately 5% in invasive lobular carcinoma versus 20% in invasive ductal carcinoma, revealing molecular-level biological heterogeneity between these two predominantly ER-positive entities (Al Dlali et al., 2025).

The prognostic significance of GATA3 mutations represents one of the most actively debated questions in the field, with conclusions showing high context dependency. Evidence for adverse prognosis is substantial: in metastatic HR + breast cancer receiving endocrine monotherapy, GATA3 mutation carriers had shorter survival (Waks et al., 2022), and in broader ER-positive cohorts, these mutations have been identified as independent poor prognostic factors (Hruschka et al., 2020). However, opposing findings challenge a blanket unfavorable interpretation. Mutations specifically affecting the second zinc finger domain—which alter the protein’s phase separation behavior and reshape the transcriptome—are paradoxically associated with favorable prognosis and slowed tumor growth (Chen et al., 2025). This observation, however, is primarily based on a single mechanistic study utilizing cell line models and limited clinical data; independent validation in large, well-annotated clinical cohorts is warranted before definitive conclusions can be drawn. This contradictory prognostic landscape strongly suggests that different mutation classes possess fundamentally distinct biological functions. The field currently lacks a functional classification framework capable of stratifying GATA3 mutations by their mechanistic consequences, representing a critical gap that limits their clinical utility for prognostic assessment and therapeutic decision-making. Such a framework would ideally integrate mutation location (e.g., zinc finger 1 vs. zinc finger two domain), impact on DNA-binding capacity and phase separation dynamics, and resultant transcriptomic alterations, enabling clinically actionable subclassification of GATA3-mutant breast cancers.

## 5 GATA3 and therapeutic response

### 5.1 The role of GATA3 in endocrine therapy response and resistance

GATA3 co-binds with ERα and FOXA1 at enhancer regions, synergistically maintaining the luminal phenotype and endocrine sensitivity in breast cancer cells (Kumar and Vindal, 2023). Disruption of this regulatory architecture drives resistance through multiple mechanisms. ARID1A inactivation mutations disrupt GATA3 and FOXA1 chromatin binding, transforming cells from an ER-dependent luminal phenotype to an ER-independent basal-like state resistant to the ER degrader fulvestrant (Xu et al., 2020). The 14-3-3τ protein promotes GATA3 degradation while upregulating the splice variant ERα36, ultimately leading to loss of full-length ERα expression and endocrine treatment failure (Garan et al., 2022). Adding further complexity, a common GATA3 splice mutant exhibits dual effects on proliferation—promoting or inhibiting—depending on the hormonal context, suggesting that the impact of specific mutations on endocrine therapy is not predictable from mutation status alone (Hruschka et al., 2020). Consequently, functional assays characterizing mutation-specific transcriptional outputs, or the development of mutation-class-specific biomarkers, may be required to translate GATA3 mutational information into clinically useful predictors of endocrine therapy response.

### 5.2 GATA3 and chemotherapy sensitivity

GATA3 status shows a clear inverse correlation with chemotherapy sensitivity. Patients achieving pathological complete response after neoadjuvant chemotherapy (NAC) have significantly lower GATA3 expression compared to those with residual disease (Chen et al., 2024). Mechanistically, GATA3 confers doxorubicin resistance by transcriptionally repressing the iron metabolism gene CYB5R2, thereby inhibiting doxorubicin-induced ferroptosis—explaining the poor response of GATA3-high luminal breast cancers to anthracycline-based regimens (Zhu et al., 2023). The lncRNA GATA3-AS1 has been independently confirmed as a predictor of non-response to NAC in locally advanced breast cancer (González-Woge et al., 2024). These findings position GATA3 and its associated molecules as potential biomarkers for chemotherapy patient selection and candidate targets for overcoming resistance.

### 5.3 Immune microenvironment and immunotherapy potential

Breast cancers with low or absent GATA3 expression tend to present an “immune-hot” phenotype, while GATA3-high tumors are predominantly “immune-cold.” Low GATA3 expression—particularly in TNBC—is significantly associated with upregulated PD-L1 expression, T-cell activation, and higher tumor-infiltrating lymphocyte levels (Saele et al., 2025; Chen et al., 2024). This immune activation may be partly attributable to the higher tumor mutation burden (TMB) observed in GATA3-low subtypes, enhancing tumor immunogenicity (Wang et al., 2025). These characteristics identify GATA3-low breast cancers as potential beneficiaries of immune checkpoint inhibitor therapy, suggesting that GATA3 status could serve as a predictive biomarker for immunotherapy patient stratification. However, whether GATA3 loss directly reshapes the immune microenvironment or merely co-occurs with intrinsically immunogenic phenotypes remains mechanistically unresolved and warrants investigation through conditional knockout models and single-cell transcriptomic approaches.

## 6 Conclusion and perspectives

GATA3, the master transcriptional regulator of mammary luminal cell fate, plays a central and multifaceted role throughout the pathogenesis of breast cancer. It functions not only as a cornerstone of the defining ERα-GATA3-FOXA1 transcriptional network in luminal breast cancer, where this regulatory axis is most operative, but its expression status and genetic integrity also profoundly impact tumor diagnosis, molecular subtyping, clinical prognosis, and therapeutic response across diverse molecular subtypes. Accumulating evidence delineates a dualistic nature of GATA3: its high expression, serving as a marker of a differentiated state, is generally correlated with favorable clinical outcomes, consistent with a tumor-suppressive role. Conversely, its high-frequency mutational status suggests a potential oncogenic driver function in tumor initiation and progression. In diagnostic pathology, GATA3 has been firmly established as one of the most sensitive and reliable immunohistochemical markers for identifying both primary and metastatic breast carcinomas, substantially refining pathological diagnostic accuracy (Chen et al., 2024). However, its heterogeneous expression in specific subtypes, notably TNBC, underscores its diagnostic limitations. The emergence of novel markers, particularly TRPS1 and SOX10, provides a robust solution to this challenge. TRPS1 demonstrates high sensitivity across all breast cancer subtypes, including metaplastic carcinoma and other rare histological variants where GATA3 frequently shows reduced expression (Parkinson et al., 2022), whereas SOX10 exhibits a complementary expression pattern to GATA3 in TNBC. Consequently, an immunohistochemical panel incorporating GATA3, TRPS1, and SOX10 represents the future direction for precision diagnosis, promising to significantly enhance diagnostic accuracy, especially in GATA3-negative cases (González-Woge et al., 2024). In prognostic stratification, GATA3 expression levels are strongly linked to the degree of tumor differentiation and invasive potential. High expression typically portends a more favorable prognosis, whereas its loss or downregulation is associated with enhanced aggressiveness and inferior outcome. The prognostic value of GATA3 gene mutations, however, presents a complex picture, as different mutation classes may confer disparate clinical consequences—for example, mutations affecting the second zinc finger domain are associated with favorable outcomes, whereas those in the N-terminal region may portend poorer prognosis—underscoring the necessity for future functional stratification of mutations to enable more precise risk assessment. In guiding treatment decisions, the status of GATA3 is pivotal for predicting therapeutic responses. Its high expression is linked to endocrine sensitivity and chemoresistance, while low expression correlates with chemosensitivity and an immune-activated tumor microenvironment, suggesting potential susceptibility to immunotherapy. These associations provide a rationale for tailoring multimodal treatment strategies-such as combinations of endocrine therapy, chemotherapy, and immunotherapy-based on GATA3 status. However, the clinical implementation of GATA3-based stratification requires standardized immunohistochemical scoring criteria and validation in prospective clinical trials before routine adoption. Mechanistically, GATA3, through synergistic interactions with core factors like ERα and FOXA1 and under the regulation of intricate epigenetic and non-coding RNA networks, collectively dictates the luminal phenotype and biological conduct of breast cancer cells (Kumar and Vindal, 2023).

Future research directions should focus on several key areas. Firstly, a deeper dissection of how specific GATA3 mutation types precisely alter protein function and ultimately drive oncogenesis is crucial for understanding breast cancer heterogeneity. Secondly, elucidating the role of the GATA3 regulatory network in governing the transition of tumor cells from a differentiated to a proliferative state may unveil novel therapeutic targets to intervene in disease progression. Thirdly, efforts should be directed towards standardizing multi-marker diagnostic panels that include TRPS1 for clinical use, optimizing the diagnostic workflow for metastatic breast cancer, particularly TNBC. Fourthly, a more granular understanding of the functional consequences of distinct GATA3 mutants and their differential impact on prognosis and treatment response is needed to achieve refined molecular stratification of patients. Finally, further investigation into the precise mechanisms by which GATA3 shapes the tumor immune microenvironment will be essential to evaluate its utility as a biomarker for identifying patients most likely to benefit from immunotherapy and to explore the potential of modulating GATA3 to enhance immunotherapeutic efficacy. In conclusion, ongoing, in-depth investigation into the multifaceted roles of GATA3 will continue to propel the field of breast cancer towards more sophisticated and personalized diagnostic and therapeutic paradigms. In this regard, conditional knockout models in immunocompetent hosts combined with single-cell transcriptomic profiling of GATA3-stratified tumors may offer valuable experimental frameworks to determine whether GATA3 loss causally remodels immune cell composition or merely accompanies intrinsically immunogenic phenotypes.
